# Supplementary material for: Polygenic risk for autism spectrum disorder associates with anger recognition in a neurodevelopment-focused phenome-wide scan of unaffected youths from a population-based cohort
Source: PLoS Genet. 2020 Sep 17;16(9):e1009036. doi: 10.1371/journal.pgen.1009036 (PMC7523983; doi:10.1371/journal.pgen.1009036)
Supplement: S1 Text — (DOCX) [file pgen.1009036.s001.docx]

**Supplemental results**

**Neurodevelopmental trait association**

Considering a suggestive threshold based on a false discovery rate correction accounting for the number of phenotypes tested only (N=491, false discovery rate (FDR) Q<0.05), there were one, 30, and two additional phenotypes association with genetic liability to ASD in the adult, middle, and young proband groups, respectively (S1 Table). The PNC traits SIP033 (Structural Interview for Prodromal Symptoms: “Has anyone pointed out to you that you are less emotional or connected to people than you used to be?”) and PADT_SAME_PC (Penn Age Differentiation Test: percent of correct responses to trials with no age difference (60 total face pairs)) were associated with ASD PRS in the adult (z-score=3.76, R^2^=3.84%, p=1.69x10^-4^) and young (z-score=-3.41, R^2^=1.06%, p=5.08x10^-04^) probands, respectively (Fig 1). When binned by quartiles, there was a 2.39-fold increase between highest quartile and lowest quartile of the ASD PRS distribution in the odds of being told that you are less emotional/connected to people than previously in the adult proband group (beta_q4vs.q1_=0.873, p_q4vs.q1_=0.015; Fig 1B) and a 2.22-fold reduction in the percentage of total correct responses to age differentiation trials when no age difference was present in the young proband group (beta_q4vs.q1_=0.799, p_q4vs.q1_=0.004; Fig 1D).

Eight out of 30 suggestively significant phenotypes from the middle proband group were nominally significantly correlated with PEITANG (0.002≤r^2^≤0.378, 2.20x10^-16^≤p≤0.037, S3 Table and S2 and S3 Figs). To verify that associations between these phenotypes and ASD PRS was independent of the effects of PEITANG, PEITANG PRS was included as a covariate in each model. ASD PRS was significantly associated with all 30 suggestively significant phenotypes from the middle proband group after covarying for PEITANG. Except for SIP011, these additional suggestive relationships with ASD PRS had no change in effect after covarying for PEITANG (S3 Fig). The phenotype SIP011: SIPS-PRIME SCREEN-REVISED Structured Interview for Prodromal Symptoms: I think I might feel like my mind is "playing tricks" on me was the only phenotype demonstrating a significant increase in effect coefficient after covarying for PEITANG (*i.e.*, after covarying PEITANG, ASD PRS more strongly predicting SIP011; covaried z-score=5.06, R^2^=0.948%, p=6.98x10^-7^; original z-score=3.05, R^2^=0.358%, p=0.002; z-score_difference_=-2.37, p_diff_=0.020).
