# Supplementary material for: Polygenic risk for autism spectrum disorder associates with anger recognition in a neurodevelopment-focused phenome-wide scan of unaffected youths from a population-based cohort
Source: PLoS Genet. 2020 Sep 17;16(9):e1009036. doi: 10.1371/journal.pgen.1009036 (PMC7523983; doi:10.1371/journal.pgen.1009036)
Supplement: S1 Fig — The x-axis shows correctness and response time for each facial emotion using two neurocognitive instruments: The Penn Emotion Differentiation Test Correctness and Reaction Time. Significance is indicated by * for p < 0.05. (DOCX) [file pgen.1009036.s002.docx]

**
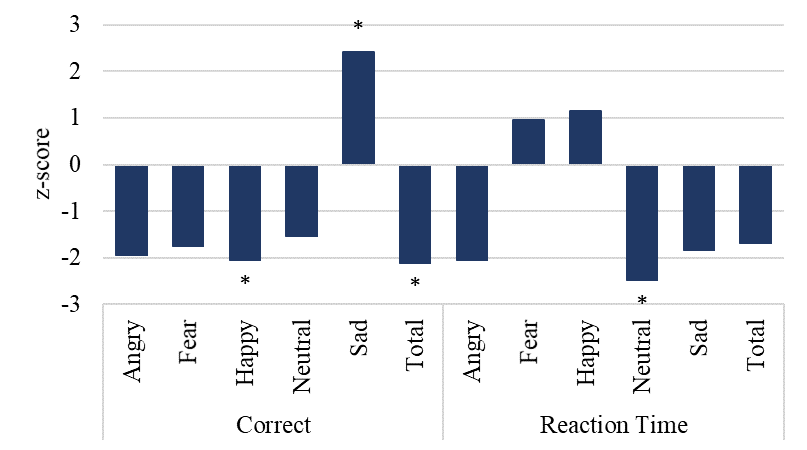
**

S1 Fig. Prediction of facial emotion differentiation capabilities of the middle proband group (ages 11 to 17) of the Philadelphia Neurodevelopmental Cohort using polygenic risk (PRS) for autism spectrum disorder. The x-axis shows correctness and response time for each facial emotion using two neurocognitive instruments: The Penn Emotion Differentiation Test. Significance is indicated by * for p < 0.05.
