# Supplementary material for: Polygenic risk for autism spectrum disorder associates with anger recognition in a neurodevelopment-focused phenome-wide scan of unaffected youths from a population-based cohort
Source: PLoS Genet. 2020 Sep 17;16(9):e1009036. doi: 10.1371/journal.pgen.1009036 (PMC7523983; doi:10.1371/journal.pgen.1009036)
Supplement: S2 Fig — Neurodevelopmental traits are labeled if the correlation with PEITANG and PRS survive multiple testing correction; these are PEIT_CR: Penn Emotion Identification Test total correct responses for all trials; GAD018: Generalized Anxiety Disorder: Did you feel any of the following physical symptoms when you worried the most: irritability (feeling easily annoyed)?; PHB012: Specific Phobia: Thinking about all of the time that you were afraid of (insert worst fear), whether or not you actually faced it, how long did this fear last? (Days); PHB014: Specific Phobia: Thinking about all of the time that you were afraid of (insert worst fear), whether or not you actually faced it, how long did this fear last? (Months). (DOCX) [file pgen.1009036.s003.docx]

**
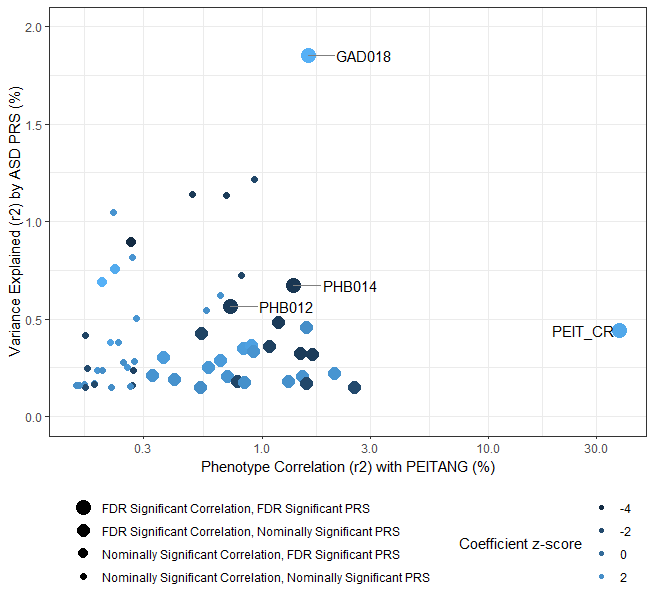
**

S2 Fig. Fifty-nine traits phenotypically correlated with PEITANG and at least nominally predicted by polygenic risk (PRS) for autism spectrum disorder (ASD) in the Philadelphia Neurodevelopmental Cohort middle proband group (age 11-17). Neurodevelopmental traits are labeled if the correlation with PEITANG and PRS survive multiple testing correction; these are PEIT_CR: Penn Emotion Identification Test total correct responses for all trials; GAD018: Generalized Anxiety Disorder: Did you feel any of the following physical symptoms when you worried the most: irritability (feeling easily annoyed)?; PHB012: Specific Phobia: Thinking about all of the time that you were afraid of (insert worst fear), whether or not you actually faced it, how long did this fear last? (Days); PHB014: Specific Phobia: Thinking about all of the time that you were afraid of (insert worst fear), whether or not you actually faced it, how long did this fear last? (Months).
