# Supplementary material for: Polygenic risk for autism spectrum disorder associates with anger recognition in a neurodevelopment-focused phenome-wide scan of unaffected youths from a population-based cohort
Source: PLoS Genet. 2020 Sep 17;16(9):e1009036. doi: 10.1371/journal.pgen.1009036 (PMC7523983; doi:10.1371/journal.pgen.1009036)
Supplement: S3 Fig — Two differences were evaluated: (1; circles) the coefficient difference between original best-fit PRS and the same PRS covaried for the effects of PEITANG (i.e., the same genome-wide significance threshold in both instances) and (2; triangles) the coefficient difference between original best-fit PRS and the best-fit PRS after covarying for the effects of PEITANG (i.e., the GWS may be different in each instance). Note that in several instances the covaried best-fit PRS is the same as the matched PRS from the original test. Filled-in shapes indicate statistically significant differences between original and covaried PRS coefficients (false discovery rate Q < 0.05). (DOCX) [file pgen.1009036.s004.docx]

**
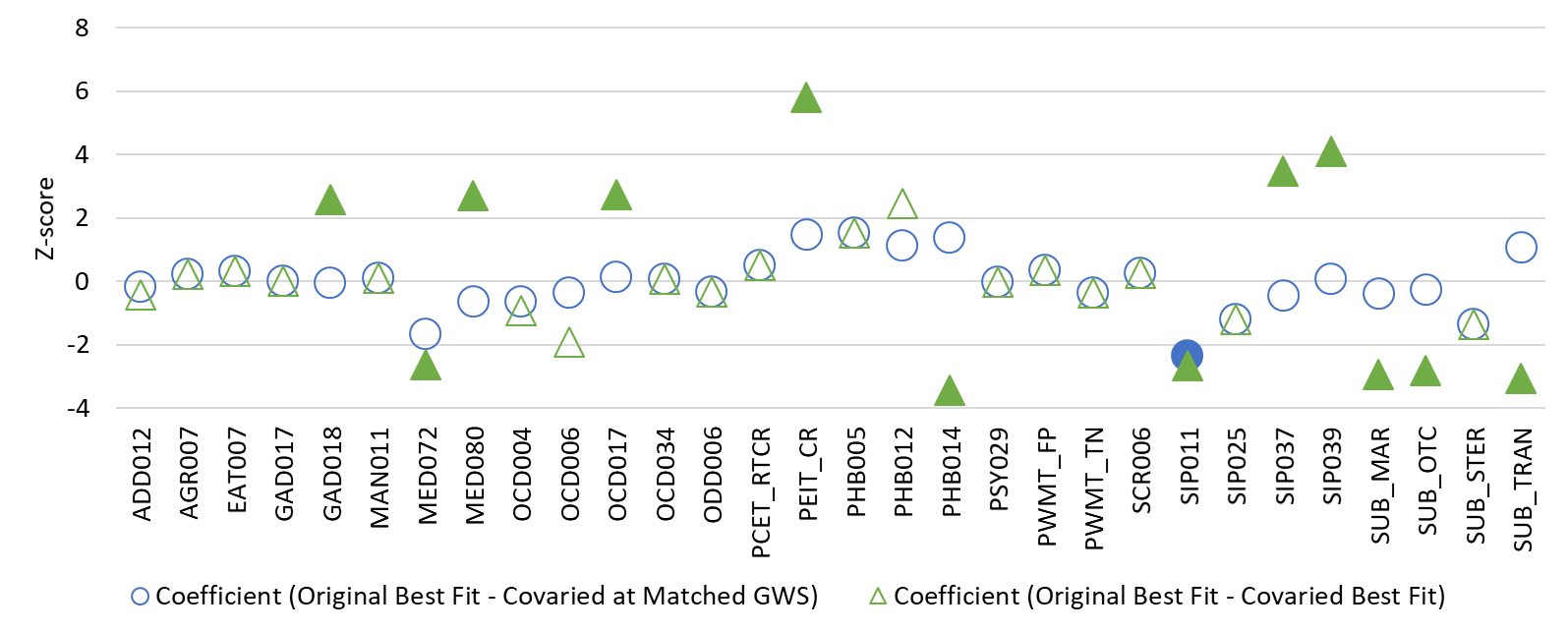
**

S3 Fig. Z-score converted difference in polygenic risk score (PRS) coefficient for 30 phenotypes suggestively predicted by polygenic risk for autism spectrum disorder in the middle proband group of the Philadelphia Neurodevelopmental Cohort. Two differences were evaluated: (1; circles) the coefficient difference between original best-fit PRS and the same PRS covaried for the effects of PEITANG (i.e., the same genome-wide significance threshold in both instances) and (2; triangles) the coefficient difference between original best-fit PRS and the best-fit PRS after covarying for the effects of PEITANG (i.e., the GWS may be different in each instance). Note that in several instances the covaried best-fit PRS is the same as the matched PRS from the original test. Filled-in shapes indicate statistically significant differences between original and covaried PRS coefficients (false discovery rate Q < 0.05).
