# Supplementary material for: Polygenic risk for autism spectrum disorder associates with anger recognition in a neurodevelopment-focused phenome-wide scan of unaffected youths from a population-based cohort
Source: PLoS Genet. 2020 Sep 17;16(9):e1009036. doi: 10.1371/journal.pgen.1009036 (PMC7523983; doi:10.1371/journal.pgen.1009036)
Supplement: S4 Fig — (A) Genetic correlation between ASD 3,199 brain imaging phenotypes from the Brain Imaging Genetics project and (B) genetic correlation between 14 brain imaging phenotypes nominally genetically correlated with ASD. (DOCX) [file pgen.1009036.s005.docx]

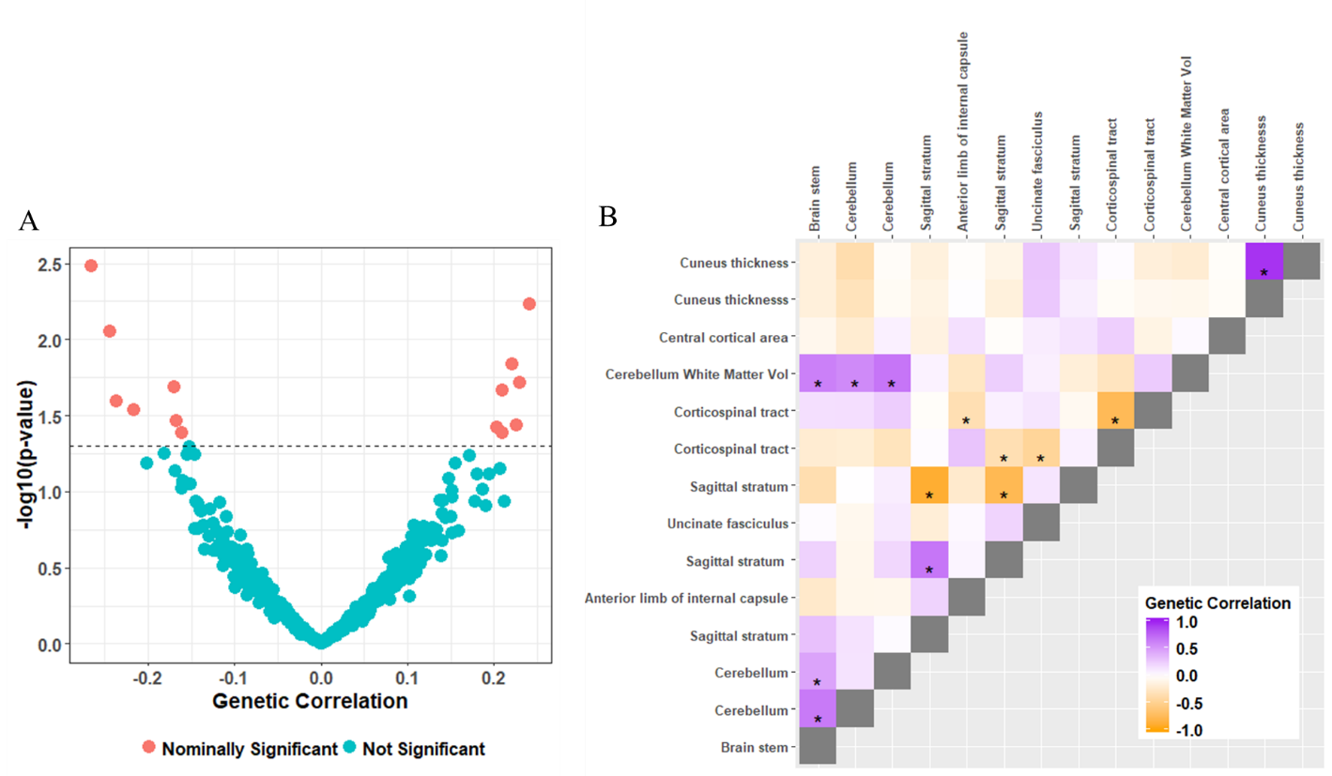


S4 Fig. Selection of brain image-derived phenotypes as covariates in the polygenic risk score analyses between autism spectrum disorder (ASD) and neuropsychiatric traits in the young. (A) Genetic correlation between ASD 3,199 brain imaging phenotypes from the Brain Imaging Genetics project and (B) genetic correlation between 14 brain imaging phenotypes nominally genetically correlated with ASD.
