# Supplementary material for: Polygenic risk for autism spectrum disorder associates with anger recognition in a neurodevelopment-focused phenome-wide scan of unaffected youths from a population-based cohort
Source: PLoS Genet. 2020 Sep 17;16(9):e1009036. doi: 10.1371/journal.pgen.1009036 (PMC7523983; doi:10.1371/journal.pgen.1009036)
Supplement: S5 Fig — (DOCX) [file pgen.1009036.s006.docx]

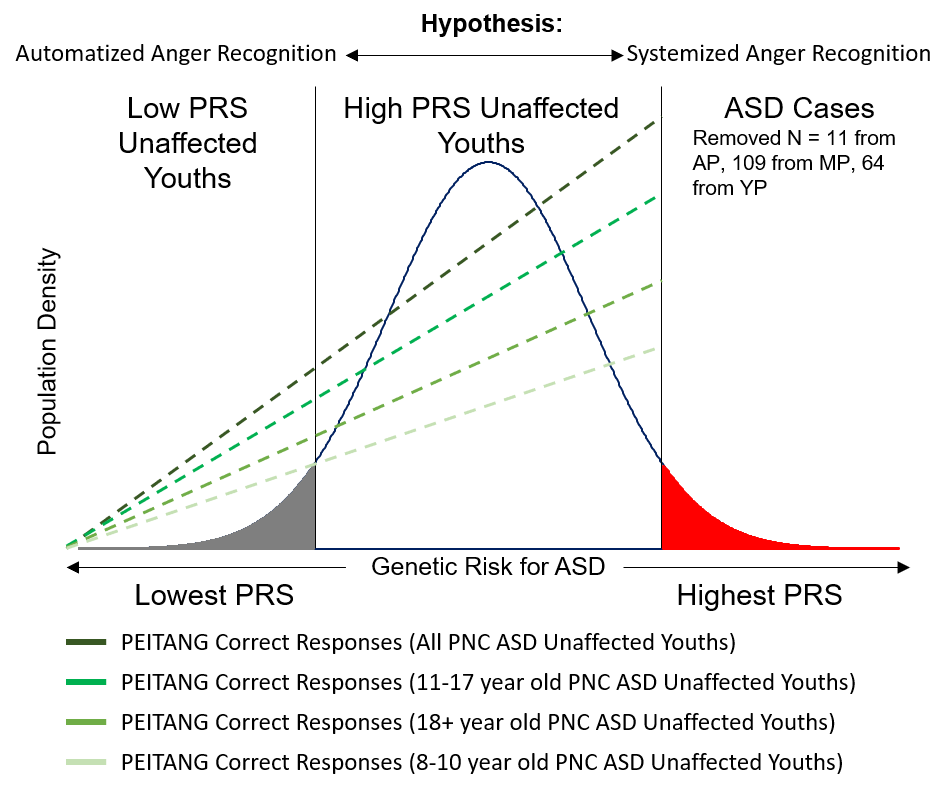


S5 Fig. Summary of the relationship between autism spectrum disorder (ASD) polygenic risk score (PRS) and anger recognition (PEITANG; Penn Emotion Identification Test correct responses to anger trials) across Philadelphia Neurodevelopmental Cohort (PNC) proband age groups.
