# Supplementary material for: Polygenic risk for autism spectrum disorder associates with anger recognition in a neurodevelopment-focused phenome-wide scan of unaffected youths from a population-based cohort
Source: PLoS Genet. 2020 Sep 17;16(9):e1009036. doi: 10.1371/journal.pgen.1009036 (PMC7523983; doi:10.1371/journal.pgen.1009036)
Supplement: S1 Table — (DOCX) [file pgen.1009036.s007.docx]

S1 Table. Phenotypes meeting suggestive threshold for significant prediction by polygenic risk for autism spectrum disorder in the young (YP), middle (MP), and adult (AP) proband groups.

| Proband | Phenotype | Description | GWS Threshold | PRS R^2^ (%) | z-score | p-value |
| --- | --- | --- | --- | --- | --- | --- |
| AP | SIP033 | SIPS- Structured Interview for Prodromal Symptoms: Has anyone pointed out to you that you are less emotional or connected to people than you used to be? | 0.00145 | 3.84 | 3.761 | 1.69E-04 |
| MP | PEITANG | PEIT: Number of Correct Responses to Anger Trials, by genus | 0.4487 | 1.056 | 5.282 | 1.38E-07 |
| MP | SUB_TRAN | Tranquilizer use endorsed | 0.3645 | 0.779 | -3.962 | 7.73E-05 |
| MP | EAT007 | Eating Disorder: Has there been a time when your eating was out of control - you'd eat a large amount of food in a short period of time and could not stop yourself? | 0.0105 | 1.144 | 3.929 | 8.53E-05 |
| MP | OCD034 | Obsessive Compulsive Disorder: Did you stay home from school/work because of your behaviors/thoughts? | 0.0009 | 5.592 | 3.909 | 9.25E-05 |
| MP | GAD018 | Generalized Anxiety Disorder: Did you feel any of the following physical symptoms when you worried the most: irritability (feeling easily annoyed)? | 0.4948 | 1.689 | 3.788 | 1.52E-04 |
| MP | SIP025 | SIPS- PRIME SCREEN-REVISED Structured Interview for Prodromal Symptoms: I think that I may hear my own thoughts being said out loud. | 0.0713 | 0.61 | 3.698 | 2.22E-04 |
| MP | OCD004 | Obsessive Compulsive Disorder: Have you ever been bothered by thoughts that don't make sense to you, that come over and over again and won't go away, such as fear that you would do something/say something bad without intending to? | 0.0164 | 1.082 | 3.685 | 2.29E-04 |
| MP | PSY029 | Psychosis: Have you ever seen visions or seen things which other people could not see? | 0.0018 | 1.346 | 3.671 | 2.41E-04 |
| MP | MED080 | Migraine & Recurrent Headaches: Do your headaches make it hard for you to do your school work or other things you want(s) to do? | 0.3953 | 1.534 | 3.596 | 3.23E-04 |
| MP | PEIT_CR | PEIT: Total Correct Responses for All Test Trials, by genus | 0.4487 | 0.454 | 3.475 | 5.59E-04 |
| MP | MED072 | Childhood: Was any part of your development abnormal in any way? For example, did you walk or talk later than other children? | 0.1201 | 0.669 | 3.387 | 7.06E-04 |
| MP | MAN011 | Mania/ Hypomania: If yes, On the days you felt this way, how much of the day did it last? | 0.04175 | 1.573 | -3.397 | 7.20E-04 |
| MP | ADD012 | Attention Deficit Disorder: Did you often have problems following instructions and often fail to finish school, work, or other things you meant to get done? | 0.0021 | 0.648 | 3.349 | 8.12E-04 |
| MP | PCET_RTCR | PCET: Median Response Time for Correct Responses | 0.0068 | 0.41 | -3.278 | 0.001 |
| MP | PHB014 | Specific Phobia: Thinking about all of the time that you were afraid of (insert worst fear), whether or not you actually faced it, how long did this fear last? (Months) | 0.4704 | 0.65 | -3.248 | 0.001 |
| MP | PWMT_TN | PWMT: Number of Correct Responses to Foil Words (TN) | 0.0006 | 0.395 | 3.242 | 0.001 |
| MP | PWMT_FP | PWMT: Number of Incorrect Responses to Foil Words (FP) | 0.0006 | 0.395 | -3.242 | 0.001 |
| MP | OCD017 | Obsessive Compulsive Disorder: Have you ever had to do something over and over again - that would have made you feel really nervous if you couldn't do it, like: doing things over and over again at bedtime, like arranging the pillows, sheets, or other things? | 0.0351 | 1.224 | 3.23 | 0.001 |
| MP | SUB_STER | Steroid use endorsed | 0.0248 | 3.921 | 3.186 | 0.001 |
| MP | AGR007 | Agoraphobia: Looking at this card, have you ever been very nervous or afraid of: traveling in a car? | 0.00985 | 3.45 | -3.165 | 0.002 |
| MP | SUB_OTC | Over the counter substance use endorsed | 0.0001 | 1.101 | -3.158 | 0.002 |
| MP | SCR006 | General Probes: Are you currently taking medication because of your emotions and/or behaviors? | 0.0024 | 0.702 | 3.128 | 0.002 |
| MP | PHB012 | Specific Phobia: Thinking about all of the time that you were afraid of (insert worst fear), whether or not you actually faced it, how long did this fear last? (Days) | 0.4222 | 0.613 | -3.072 | 0.002 |
| MP | OCD006 | Obsessive Compulsive Disorder: Have you ever been bothered by thoughts that don't make sense to you, that come over and over again and won't go away, such as forbidden/bad thoughts? | 0.0116 | 1.227 | 3.058 | 0.002 |
| MP | SIP011 | SIPS- PRIME SCREEN-REVISED Structured Interview for Prodromal Symptoms: I think I might feel like my mind is "playing tricks" on me. | 0.03985 | 0.358 | 3.047 | 0.002 |
| MP | SIP037 | SIPS- Structured Interview for Prodromal Symptoms: EXPRESSION OF EMOTION: Severity Scale | 0.4248 | 0.361 | 3.038 | 0.002 |
| MP | SIP039 | SIPS- Structured Interview for Prodromal Symptoms: Within the past 6 months, are you having a harder time getting normal activities done? | 0.0005 | 1.165 | -2.978 | 0.003 |
| MP | SUB_MAR | Marijuana use endorsed | 0.0001 | 3.72 | -2.977 | 0.003 |
| MP | ODD006 | Oppositional Defiant Disorder: Were you often irritable or grouchy, or did you often get angry because you thought that things were unfair? | 0.0021 | 0.469 | 2.967 | 0.003 |
| MP | PHB005 | Specific Phobia: Looking at this card, have you ever been very nervous or afraid of doctors, needles, or blood? | 1 | 0.46 | -2.959 | 0.003 |
| MP | GAD017 | Generalized Anxiety Disorder: Did you feel any of the following physical symptoms when you worried the most: concentration problems (trouble focusing or paying attention)? | 0.0001 | 1.062 | -2.943 | 0.003 |
| YP | PADT_SAME_CR | PADT: Number of Correct Responses to Test Trials with No Age Difference, by genus | 0.0986 | 1.11 | -3.487 | 5.08E-04 |
| YP | PADT_SAME_PC | PADT: Percent Correct Responses to Test Trials with No Age Difference, by genus | 0.0986 | 1.11 | -3.487 | 5.08E-04 |
| GWS Threshold: p-value applied to the autism spectrum disorder GWAS for calculating polygenic risk scores  PRS R^2^: measure of phenotype variance explained by association with ASD PRS  z-score: normalized measure of PRS effect magnitude relative to its standard error (z = beta/se)  p-value: test statistic for the ASD PRS 🡪 phenotype estimate | | | | | | |
