# Supplementary material for: Polygenic risk for autism spectrum disorder associates with anger recognition in a neurodevelopment-focused phenome-wide scan of unaffected youths from a population-based cohort
Source: PLoS Genet. 2020 Sep 17;16(9):e1009036. doi: 10.1371/journal.pgen.1009036 (PMC7523983; doi:10.1371/journal.pgen.1009036)
Supplement: S2 Table — (DOCX) [file pgen.1009036.s008.docx]

Table S2. Spearman’s correlation (*r^2^*) and significance (*p*-value) between PEITANG and other measures recorded by the Penn Emotion Identification Test (PEIT).

| Trait | Emotion | Measure | r^2^ | p-value |
| --- | --- | --- | --- | --- |
| PEIT_CR | All | Correct | 0.378 | 2.20E-16 |
| PEIT_CRT | All | Reaction Time | 0.005 | 3.37E-04 |
| PEITANGRT | Anger | Reaction Time | 0.012 | 2.87E-08 |
| PEITFEAR | Fear | Correct | 0.007 | 1.86E-05 |
| PEITFEARRT | Fear | Reaction Time | 0.002 | 3.79E-02 |
| PEITHAP | Happiness | Correct | 0.022 | 1.85E-14 |
| PEITHAPRT | Happiness | Reaction Time | 0.008 | 2.96E-06 |
| PEITNOE | Neutral | Correct | 0.008 | 6.39E-06 |
| PEITNOERT | Neutral | Reaction Time | 0.001 | 8.00E-02 |
| PEITSAD | Sad | Correct | 0.20 | 7.46E-13 |
| PEITSADRT | Sad | Reaction Time | 0.003 | 1.02E-02 |
