# Supplementary material for: Polygenic risk for autism spectrum disorder associates with anger recognition in a neurodevelopment-focused phenome-wide scan of unaffected youths from a population-based cohort
Source: PLoS Genet. 2020 Sep 17;16(9):e1009036. doi: 10.1371/journal.pgen.1009036 (PMC7523983; doi:10.1371/journal.pgen.1009036)
Supplement: S3 Table — (DOCX) [file pgen.1009036.s009.docx]

S3 Table. Traits significantly correlated (r^2^ and correlation p) with PEITANG in the middle proband group of the Philadelphia Neurodevelopmental Cohort and at least nominally associated with polygenic risk (PRS) for autism spectrum disorder (PRS R^2^, z-score, and PRS p).

| *Phenotype* | *Description* | *r^2^ (%)* | *Correlation p* | *PRS R^2^ (%)* | *z-score* | *PRS p* |
| --- | --- | --- | --- | --- | --- | --- |
| ADD015 | Attention Deficit Disorder: Did you often have trouble making plans, doing things that had to be done in a certain kind of order, or that had a lot of different steps? | 0.232 | 0.015 | 0.381 | 2.44 | 0.015 |
| DEP001 | Depression: Has there ever been a time when you felt sad or depressed most of the time? | 0.197 | 0.024 | 0.237 | 2.001 | 0.045 |
| GAD015 | Generalized Anxiety Disorder: Did you feel any of the following physical symptoms when you worried the most: restlessness? | 0.651 | 0.007 | 0.624 | 2.266 | 0.023 |
| GAD017 | Generalized Anxiety Disorder: Did you feel any of the following physical symptoms when you worried the most: concentration problems (trouble focusing or paying attention)? | 0.493 | 0.02 | 1.141 | -3.02 | 0.003 |
| GAD018 | Generalized Anxiety Disorder: Did you feel any of the following physical symptoms when you worried the most: irritability (feeling easily annoyed)? | 1.595 | 2.74E-05 | 1.854 | 3.891 | 9.97E-05 |
| LNB_FP0 | LNB: Number of Incorrect Responses to 0-Back Trials (FP) | 0.533 | 2.03E-04 | 0.148 | 1.962 | 0.05 |
| LNB_MRTC | LNB: Mean of the Median Response Time for Correct Responses for 1-Back (TP) and for 2-Back (TP) Trials | 0.262 | 0.009 | 0.155 | 2.005 | 0.045 |
| LNB_RTC1 | LNB: Median Response Time for Correct Responses 1-Back Trials (TP) | 0.215 | 0.018 | 0.15 | 1.97 | 0.049 |
| MAN006 | Mania/ Hypomania: Have you ever had a time when you felt like you could do almost anything? | 0.244 | 0.012 | 0.277 | 2.057 | 0.04 |
| MED003 | Medical: Weight: lb. | 0.83 | 1.62E-05 | 0.352 | 2.827 | 0.005 |
| MED072 | Childhood: Was any part of your development abnormal in any way? For example, did you walk or talk later than other children? | 0.225 | 0.016 | 0.757 | 3.583 | 0.00034 |
| MED248 | Speech problem - Do/did you have this problem? | 0.91 | 1.17E-06 | 0.333 | 2.348 | 0.019 |
| MED273 | Learning problem - Do/did you have this problem? | 0.17 | 0.036 | 0.248 | -1.986 | 0.047 |
| MED803C | Is it (ear/nose/throat problems) current (within last 6 months)? | 0.811 | 0.005 | 0.724 | -2.245 | 0.025 |
| MED809 | Do you or did you have any of the following problems? - Infectious Disease - an illness from a virus, bacteria (Has Problem?) | 0.541 | 1.81E-04 | 0.426 | -2.394 | 0.017 |
| MED815C | Is it (pulmonary condition) current (within last 6 months)? | 0.92 | 0.024 | 1.219 | -2.153 | 0.031 |
| OCD007 | Obsessive Compulsive Disorder: Have you ever been bothered by thoughts that don't make sense to you, that come over and over again and won't go away, such as need for symmetry/exactness? | 0.277 | 0.008 | 0.502 | 2.42 | 0.016 |
| OCD026 | Obsessive Compulsive Disorder: About how much of a typical day did you spend thinking these thoughts or engaging in these behaviors? (Minutes) (in response to "Do you feel the need to do things just right (like they have to be perfect)?") | 1.565 | 1.86E-04 | 0.458 | 2.021 | 0.044 |
| PAN001 | Panic Disorder: Have you ever had an attack like this? | 0.272 | 0.008 | 0.282 | 2.11 | 0.035 |
| PCPT_L_TPRT | PCPT: Median Response Time for Correct Responses to Letter Trials (TP) | 0.329 | 0.003 | 0.209 | 2.337 | 0.02 |
| PCPT_N_TPRT | PCPT: Median Response Time for Correct Responses to Number Trials (TP) | 0.893 | 1.37E-06 | 0.366 | 3.092 | 0.002 |
| PCPT_T_TPRT | PCPT: Median Response Time for Correct Responses to Number Trials (TP) and Letter Trials (TP) | 0.657 | 3.50E-05 | 0.288 | 2.74 | 0.006 |
| PEDT_A | PEDT: Total Correct Responses for All Test Trials, by genus | 1.571 | 1.36E-10 | 0.171 | -2.116 | 0.034 |
| PEDT_HAP_CR | PEDT: Number of Correct Responses to Happy Trials | 0.267 | 0.008 | 0.16 | -2.048 | 0.041 |
| PEDT_PC | PEDT: Percent of Correct Responses for All Test Trials, by genus | 2.547 | 2.22E-16 | 0.147 | -1.965 | 0.05 |
| PEDT_SAD_CR | PEDT: Number of Correct Responses to Sad Trials, by genus | 2.079 | 1.43E-13 | 0.22 | 2.404 | 0.016 |
| PEDT_SAME_RTCR | PEDT: Median Response Time for Correct Responses to Test Trials with Neutral Difference | 0.271 | 0.008 | 0.237 | -2.477 | 0.013 |
| PEIT_CR | PEIT: Total Correct Responses for All Test Trials, by genus | 37.83 | 2.22E-16 | 0.442 | 3.419 | 0.001 |
| PEITFEAR | PEIT: Number of Correct Responses to Fear Trials, by genus | 0.7 | 1.86E-05 | 0.207 | 2.33 | 0.02 |
| PEITFEARRT | PEIT: Median Response Time for Correct Fear Trial Responses, by genus | 0.165 | 0.038 | 0.149 | -1.976 | 0.048 |
| PEITHAPRT | PEIT: Median Response Time for Correct Happy Trial Responses, by genus | 0.834 | 2.96E-06 | 0.174 | 2.156 | 0.031 |
| PEITNOE | PEIT: Number of Correct Responses to Neutral Trials, by genus | 0.778 | 6.39E-06 | 0.18 | -2.173 | 0.03 |
| PFMT_FP | PFMT: Number of Incorrect Responses to Foil Faces (FP) | 1.497 | 3.66E-10 | 0.208 | -2.334 | 0.02 |
| PFMT_TN | PFMT: Number of Correct Responses to Foil Faces (TN) | 1.497 | 3.66E-10 | 0.208 | 2.334 | 0.02 |
| PHB012 | Specific Phobia: Thinking about all of the time that you were afraid of (insert worst fear), whether or not you actually faced it, how long did this fear last? (Weeks) | 0.721 | 0.001 | 0.564 | -2.939 | 0.003 |
| PHB014 | Specific Phobia: Thinking about all of the time that you were afraid of (insert worst fear), whether or not you actually faced it, how long did this fear last? (Months) | 1.366 | 3.55E-06 | 0.672 | -3.251 | 0.001 |
| PLOT_OFF | PLOT: Total Positions Off for All Test Trials, by genus | 1.301 | 5.90E-09 | 0.182 | 2.174 | 0.03 |
| PLOT_PC | PLOT: Percent Correct Responses for All Test Trials, by genus | 1.469 | 6.16E-10 | 0.323 | -2.92 | 0.004 |
| PLOT_TC | PLOT: Total Correct Responses for All Test Trials, by genus | 1.666 | 4.37E-11 | 0.317 | -2.891 | 0.004 |
| PSY104 | Psychosis: At the time that you were having (insert symptoms), were you also depressed (Feeling very sad)? | 0.567 | 0.035 | 0.543 | 2.075 | 0.038 |
| PTD006 | Post-Traumatic Stress: Have you ever been threatened with a weapon? | 0.22 | 0.017 | 1.049 | 2.349 | 0.019 |
| PTD021 | Post-Traumatic Stress: When did (insert worst event name) occur? (Month) | 0.692 | 0.023 | 1.135 | -2.913 | 0.004 |
| PWMT_KIWRD_RTC | PWMT: Median Response Time for Total Correct Test Trial Responses | 0.151 | 0.047 | 0.158 | 2.031 | 0.042 |
| PWMT_TPRT | PWMT: Median Response Time for Correct Responses to Target Words (TP) | 0.254 | 0.01 | 0.253 | 2.574 | 0.01 |
| SCR136 | General Probes: Was there ever a time when you or someone else thought you needed help or treatment for any problems we haven't discussed? | 0.266 | 0.009 | 0.816 | 2.367 | 0.018 |
| SEP509 | Separation Anxiety: When you knew that you were going to be away from home or (attachment figure(s)), did you get very upset and worry (e.g., when you learned (attachment figure(s)) were going on an upcoming trip or night out)? | 0.165 | 0.039 | 0.415 | -2.446 | 0.014 |
| SIP004 | SIPS- PRIME SCREEN-REVISED: I think that I might be able to predict the future. | 0.156 | 0.045 | 0.161 | 2.052 | 0.04 |
| SIP007 | SIPS- PRIME SCREEN-REVISED Structured Interview for Prodromal Symptoms: I think I may get confused at times whether something I experience or perceive may be real or may be just part of my imagination or dreams. | 0.364 | 0.002 | 0.301 | 2.793 | 0.005 |
| SIP008 | SIPS- PRIME SCREEN-REVISED Structured Interview for Prodromal Symptoms: I have thought that it might be possible that other people can read my mind, or that I can read others' minds | 0.187 | 0.028 | 0.237 | 2.473 | 0.013 |
| SIP010 | SIPS- PRIME SCREEN-REVISED Structured Interview for Prodromal Symptoms: I believe that I have special natural or supernatural gifts beyond my talents and natural strengths. | 0.581 | 1.08E-04 | 0.252 | 2.553 | 0.011 |
| SIP012 | SIPS- PRIME SCREEN-REVISED Structured Interview for Prodromal Symptoms: I have had the experience of hearing faint or clear sounds of people or a person mumbling or talking when there is no one near me. | 0.164 | 0.04 | 0.164 | 2.06 | 0.04 |
| SIP013 | SIPS- PRIME SCREEN-REVISED Structured Interview for Prodromal Symptoms: I think that I may hear my own thoughts being said out loud. (agree/disagree ratings) | 0.214 | 0.019 | 0.379 | 3.133 | 0.002 |
| SIP025 | SIPS- PRIME SCREEN-REVISED Structured Interview for Prodromal Symptoms: I think that I may hear my own thoughts being said out loud. (frequency of events) | 0.196 | 0.037 | 0.689 | 3.932 | 8.67E-05 |
| SIP030 | SIPS- Structured Interview for Prodromal Symptoms: Changes in speech, disorganized communication, tangential speech Severity Scale | 0.409 | 0.001 | 0.191 | 2.223 | 0.026 |
| SUB_ALC | Alcohol use endorsed | 1.176 | 4.22E-06 | 0.482 | -2.945 | 0.003 |
| SUB_COC | Cocaine use endorsed | 1.072 | 1.13E-05 | 0.36 | -2.543 | 0.011 |
| SUB_TRAN | Tranquilizer use endorsed | 0.264 | 0.03 | 0.893 | -4.019 | 6.09E-05 |
| VOLT_SVTFP | VOLT: Number of Incorrect Responses to Foil Shapes (FP) | 0.181 | 0.03 | 0.166 | -2.079 | 0.038 |
| VOLT_SVTTN | VOLT: Number of Correct Responses to Foil Shapes (TN) | 0.182 | 0.029 | 0.169 | 2.099 | 0.036 |
| r^2^: Spearman’s correlation between PEITANG and the indicated phenotype  correlation p: test statistic for Spearman’s correlation  PRS R^2^: measure of phenotype variance explained by association with ASD PRS  z-score: normalized measure of PRS effect magnitude relative to its standard error (z = beta/se)  p-value: test statistic for the ASD PRS 🡪 phenotype estimate | | | | | | |
