# Supplementary material for: Polygenic risk for autism spectrum disorder associates with anger recognition in a neurodevelopment-focused phenome-wide scan of unaffected youths from a population-based cohort
Source: PLoS Genet. 2020 Sep 17;16(9):e1009036. doi: 10.1371/journal.pgen.1009036 (PMC7523983; doi:10.1371/journal.pgen.1009036)
Supplement: S4 Table — (DOCX) [file pgen.1009036.s010.docx]

S4 Table. Samples size and SNP-based observed-scale heritability (h^2^) for genome-wide association study summary statistics used in this study. PGC: Psychiatric Genomics Consortium; Social Science Genetic Association Consortium; BIG: Brain Imaging Genetics

| **Trait** | **Abbreviation** | **Sample Size** | **h^2^ z-score** | **Source** | **Reference** |
| --- | --- | --- | --- | --- | --- |
| Autism spectrum disorder | ASD | 18,382 cases and 27,969 controls | 11.51 | PGC | (1) |
| Attention deficit hyperactivity disorder | ADHD | 20,183 cases and 35,191 controls | 15.77 | PGC | (15) |
| Schizophrenia | SCZ | 36,989 cases and 113,075 controls | 21.62 | PGC | (21) |
| Anorexia nervosa | AN | 3,495 cases and 10,982 controls | 6.27 | PGC | (16) |
| Major depressive disorder | MDD | 59,851 cases and 113,154 controls | 15.74 | PGC | (18) |
| Bipolar disorder | BIP | 20,352 cases and 31,358 controls | 15.62 | PGC | (17) |
| Tourette syndrome | TS | 4,819 cases and 9,488 controls | 8.14 | PGC | (19) |
| Obsessive compulsive disorder | OCD | 2,688 cases and 7,037 controls | 6.36 | PGC | (20) |
| Educational attainment | EA | 766,345 | 41 | SSGAC | (22) |
| Mean diffusion tensor mode (MO) in sagittal stratum (left) on fractional anisotropy skeleton (from dMRI data) | - | 7,532 | 4.5 | BIG | (14) |
| Mean orientation dispersion index (DO) in sagittal stratum (left) on fractional anisotropy skeleton (from dMRI data) | - | 7,532 | 4.08 | BIG | (14) |
| Weighted-mean orientation dispersion index (DO) in left corticospinal tract (from dMRI data) | - | 7,532 | 5.17 | BIG | (14) |
| Weighted-mean fractional anisotropy in tract left corticospinal tract (from dMRI data) | - | 7,532 | 4.48 | BIG | (14) |
| Left hemisphere cuneus gyrus thickness (from Destrieux Atlas) | - | 9,707 | 4.16 | BIG | (14) |
| Volume of grey matter in Vermis X Cerebellum | - | 8,411 | 4.88 | BIG | (14) |
| Left hemisphere cuneus thickness (from DKT Atlas) | - | 9,707 | 5 | BIG | (14) |
| Volume of grey matter in Left X Cerebellum | - | 8,411 | 4.12 | BIG | (14) |
| Mean L1 in sagittal stratum (right) on fractional anisotropy skeleton (from dMRI data) | - | 7,532 | 4 | BIG | (14) |
| Volume of grey matter in Brain-Stem | - | 8,411 | 4.38 | BIG | (14) |
| Right hemisphere central sulcus area (from Destrieux Atlas) | - | 9,707 | 5.77 | BIG | (14) |
| Mean L3 in uncinate fasciculus (left) on fractional anisotropy skeleton (from dMRI data) | - | 7,532 | 5.29 | BIG | (14) |
| Mean L1 in anterior limb of internal capsule (right) on fractional anisotropy skeleton (from dMRI data) | - | 7,532 | 4.63 | BIG | (14) |
| *Left cerebellar white matter volume* | - | 9,707 | 5.06 | BIG | (9) |
