# Supplementary material for: Polygenic risk for autism spectrum disorder associates with anger recognition in a neurodevelopment-focused phenome-wide scan of unaffected youths from a population-based cohort
Source: PLoS Genet. 2020 Sep 17;16(9):e1009036. doi: 10.1371/journal.pgen.1009036 (PMC7523983; doi:10.1371/journal.pgen.1009036)
Supplement: S5 Table — (DOCX) [file pgen.1009036.s011.docx]

S5 Table. Phenotypes associated with polygenic risk for autism spectrum disorder based on perceived behavior of middle age group (11 to 17 years old) informants from the Philadelphia Neurodevelopmental Cohort.

| *Phenotype* | *Description* | *GWS Threshold* | *PRS R2 (%)* | *z-score* | *p* | *FDR Q* |
| --- | --- | --- | --- | --- | --- | --- |
| ADD020 | Did you often have difficulty sitting still for more than a few minutes at a time, even after being asked to stay seated, or did you often fidget with your hands or feet or wiggle in your seat or were you "always on the go"? | 0.0152 | 0.633 | 3.27 | 1.17E-04 | 0.037 |
| ADD028 | Did family members seem upset, angry, or annoyed with you because of your difficulties? | 0.326 | 1.62 | 3.60 | 0.00 | 0.023 |
| ADD029 | Did these behaviors/inattention bother your friends? | 0.2256 | 1.66 | 3.62 | 3.39E-04 | 0.023 |
| AGR003 | Agoraphobia: Looking at this card, have you ever been very nervous or afraid of: being in an open field? | 0.00145 | 8.57 | 3.15 | 3.19E-04 | 0.045 |
| CDD026 | Conduct Disorder: How old were you the first time you did these (list behaviors)? (Age) | 0.0141 | 3.66 | -3.13 | 0.002 | 0.045 |
| DEP009 | When did you feel the most (sad, grouchy, irritable, in a bad mood, had trouble having fun)? | 0.00195 | 1.12 | 3.27 | 0.002 | 0.037 |
| DEP014 | Depression: During this time, did you have trouble sitting still or feel like you had to keep moving around OR did you move or think more slowly than usual? | 0.0006 | 3.63 | -3.26 | 0.001 | 0.037 |
| DEP017 | Depression: During this time, did you blame yourself for bad things that happened or feel like you didn't really matter? | 0.406 | 3.28 | 3.10 | 0.001 | 0.045 |
| MAN009 | Mania/ Hypomania: Was this different from how you usually are? (Follow up to "Has there ever been a time when you felt unusually grouchy, cranky, or irritable; when the smallest things would make you really mad?" | 0.4759 | 2.69 | 3.32 | 0.002 | 0.037 |
| MAN026 | Mania/ Hypomania: During this time when you felt the most (too happy/excited/grouchy/energetic) did you become more interested in sex or more sexually active? | 0.01295 | 9.41 | -3.62 | 9.04E-04 | 0.023 |
| ODD025 | Oppositional Defiant Disorder: How old were you the first time you did these? (Age) (in response to Did you stay home or were you sent home from school/work because of your behavior?) | 0.1177 | 1.28 | -3.53 | 2.95E-04 | 0.025 |
| PAN012 | Panic Disorder: How much did having these [panic] attacks upset or bother you? | 0.05175 | 18.27 | 3.28 | 4.35E-04 | 0.045 |
| PHB012 | Specific Phobia: Thinking about all of the time that you were afraid of (insert worst fear), whether or not you actually faced it, how long did this fear last? (Days) | 0.0008 | 1.14 | 3.77 | 0.002 | 0.023 |
| PHB014 | Specific Phobia: Thinking about all of the time that you were afraid of (insert worst fear), whether or not you actually faced it, how long did this fear last? (Months) | 0.014 | 1.20 | -3.86 | 1.73E-04 | 0.023 |
| PHB024A | Specific Phobia: How old were you the first time you had this fear of (insert worst fear)? (Age) | 0.3757 | 2.00 | -3.14 | 0.002 | 0.045 |
| PSY003 | Psychosis: How many voices did you hear? | 0.00015 | 19.40 | -3.57 | 7.38E-04 | 0.037 |
| PSY017 | Psychosis: How long was the longest time that this [hearing voices] lasted? (Estimate duration; can be intermittent over this time) (Years) | 0.0002 | 20.70 | -3.79 | 3.58E-04 | 0.023 |
| PSY054 | Psychosis: How many times have you smelled strange odors like this that other people couldn't smell? | 0.00155 | 48.68 | 4.48 | 2.07E-04 | 0.023 |
| PTD007 | Post-Traumatic Stress: Have you ever been in a bad accident? | 0.0592 | 1.23 | -3.67 | 2.45E-04 | 0.023 |
| PTD009 | Post-Traumatic Stress: Have you ever been very upset by seeing a dead body or by seeing pictures of the dead body of somebody you knew well? | 0.0019 | 0.809 | 3.14 | 0.002 | 0.045 |
| SCR004 | General Probes: How long did you see someone [a counselor] in total? | 0.00825 | 1.43 | 3.28 | 0.001 | 0.037 |
| SCR006 | General Probes: Are you currently taking medication because of your emotions and/or behaviors? | 0.3954 | 0.667 | 3.06 | 0.002 | 0.048 |
| SIP036 | SIPS- Structured Interview for Prodromal Symptoms: Changes in perception of self, others, or the world in general: How long has it been since you first had this experience? | 0.01055 | 46.96 | -3.98 | 0.001 | 0.042 |
| SIP039 | SIPS- Structured Interview for Prodromal Symptoms: Within the past 6 months, are you having a harder time getting normal activities done? | 0.0006 | 1.07 | -3.36 | 7.77E-04 | 0.037 |
| GWS Threshold: p-value applied to the autism spectrum disorder GWAS for calculating polygenic risk scores  PRS R^2^: measure of phenotype variance explained by association with ASD PRS  z-score: normalized measure of PRS effect magnitude relative to its standard error (z = beta/se)  p-value: test statistic for the ASD PRS 🡪 phenotype estimate  FDR Q: false discovery rate for each p-value | | | | | | |
