# Supplementary material for: Polygenic risk for autism spectrum disorder associates with anger recognition in a neurodevelopment-focused phenome-wide scan of unaffected youths from a population-based cohort
Source: PLoS Genet. 2020 Sep 17;16(9):e1009036. doi: 10.1371/journal.pgen.1009036 (PMC7523983; doi:10.1371/journal.pgen.1009036)
Supplement: Attachment — Submitted filename: 2020_07_20_response_to_review.docx [file pgen.1009036.s012.docx]

Dear Editor,

We thank you for facilitating the review of our article titled **Polygenic risk for autism spectrum disorder associates with anger recognition in a neurodevelopment-focused phenome-wide scan of unaffected youths from a population-based cohort**. We have carefully considered the comments from Reviewers and provide our responses below. We look forward to hearing from you about the progression and disposition of our article.

Reviewer #1: Wendt et al conducted very interesting study which examined possible association between polygenic risk score of autism spectrum disorder and various neurodevelopmental domains using large sample size (N=4,309). The manuscript is concise and the results were statistically robust. I believe that the conclusion drawn is very attractive for readers.

Major comments:

Author summary

The last sentence sounds a bit strong since the subjects analyzed in this study are lack of ASD diagnosis.

Author response: We have revised the last sentence (lines 51-52) of the Author Summary section to read “This finding is consistent with existing theories of the relationship between ASD genetic liability and a person’s ability to build generalizable and impulse driven models for responding to social phenomena.”

Results:
1. The brief summary of results for measured neurodevelopmental domains should be described in the main text.

Author response: Introduction to the PNC neurodevelopmental domains has been provided in the beginning of the Results section (lines 95-111) and the complete description has been moved from Supplementary to the Methods section (lines 318-345).

2. I do not deny in using Best-fit PRS methods, but it is known that this method may cause over-fitting. Although figure 3 clearly demonstrated that association of ASD PRS and PEITANG is robust and not a type I error, the p-value threshold and the number of SNPs analyzed in best-fit model should be described somewhere (main text or figure 1).

Author response: We have included the GWS p-value threshold determined as best-fit for the PEITANG-ASD relationship and the number of SNPs included at this threshold (lines 127-128).

Discussion:
1. The authors hypothesized that it is difficult for ASD subjects to perceive positive emotion or neutral emotion. I believe that this hypothesis has been supported by many previous studies, so references should be cited.

Author response: As requested by Reviewer 2, we have reduced our discussion of systemizing theory and as such this statement is no longer in the manuscript.

2. The author also hypothesized that ASD children are familiar with anger because their caregivers have anger emotion for raising them. I do not agree with this since the target population has lack of ASD diagnosis. The authors would like to say that as they are probands for ASD subjects, they have more opportunities to see that their caregivers get angry with their siblings?

Author response: We agree with the Reviewer that this is a weakly supported discussion point. The paragraph discussing caregiver effects have been removed.

Methods:
The summary of measured neurodevelopmental domains and batteries should be described in the main text.

Author response: Information from the supplementary material has been moved to the main text to describe the clinical testing and neurocognitive battery administered to each PNC participant (lines 95-111 and lines 318-345).

Minor points

1. In the abstract, please indicate if the p-values have been corrected for multiple testing or not in the abstract.

2. Ref 15 (Demontis D, Walters RK, Martin J, Mattheisen M, Als TD, Agerbo E, et al. Discovery Of The First Genome-Wide Significant Risk Loci For ADHD. bioRxiv. 2017) has already been published in Nature Genetics in 2018.

3. In supplemental tables, please add descriptions for abbreviations, such as GWS, correlation P, PRS P.

Author response: We thank the Reviewer for these suggestions. We have made all changes accordingly.

Reviewer #2: The authors performed a phenome-wide association study the of ASD PRS on 491 neurodevelopmental phenotypes in 4,309 probands in the Philadelphia Neurodevelopmental Cohort. The results were stratified by age group and it is noted that the these individuals lack an ASD diagnosis. An positive association with between the ASD PRS and PEITANG, a quantitative trait that measures variability in the recognition of angry emotion was observed. This association was follow up through the analysis of additional brain-imaging and psychiatric polygenic scores. The aim of these analyses is to demonstrate that evidence of an ASD-specific polygenic signal by adjusting for additional PRS. This is an interesting approach, although difficult to follow at times and would benefit from the flow chart in the supplement moved to the main text.

Author response: We appreciate the Reviewer’s suggestion to improve clarity and have moved the flowchart from supplementary information to the main text.

Overall, I found this to be an interesting follow up study from Grove et al. for understanding the contribution of the autism polygenic score to behavior and psychometric traits in the general population. Overall, the manuscript is well-written and will be of interest to autism researchers studying common variation. My main criticism is that the scope of the research is narrow. For example, it is unclear why the investigators limited the PheWAS to one neurodevelopmental/psychiatric polygenic score (ASD PRS) in the NPC cohort. In my opinion, a battery of polygenic scores in the main PheWAS analysis to understand polygenic associations with NPC measurements would have been worthwhile.

Author response: We agree with the Reviewer that a large study of neurodevelopment and polygenic risk for psychiatric disorders would be of great interest to the community. This is already a rather dense study with numerous follow-up analyses of the main findings and we believe that the multiple testing burden of the proposed set of analysis is quite prohibitive given the sample size and power differences across available psychiatric disorders (e.g., major depressive disorder N>1 million versus Tourette syndrome N~14,000). Under the proposed framework, the analysis is most likely to detect results for major depression while interesting Tourette findings may be lost to multiple testing correction. We are currently working on an additional study to use multivariate statistics approach to combine a battery of psychiatric disorders and traits and analyze the latent factors obtained with respect to neurodevelopmental features. We added this future perspective in the conclusion of the study (lines 298-301).

Questions/Comments
1. Ascertainment into the PNC cohort should be in mentioned in the main text prior to the dissemination of the results. While noting the lack of ASD diagnoses is helpful, it should be more clearly stated that these patients are not enriched for any clinical diagnoses and more representative of adolescents from the general population.

Author response: A subsection has been added to the Results section (*PNC Participant Inclusion and Description*, lines 95-111) to introduce the PNC and their generally healthy status, including removal of all ASD diagnosed participants from our analysis.

2. The total number of study-wide tests is not clearly stated in the manuscript. For each PRS, it seems it all the different p-value thresholds and all were included in the PheWAS. It’s not clear why this was selected over choosing a single PRS a priori as reported in Zheutlin et al.. Similar to SCZ, ASD is highly polygenic trait and unlikely to benefit in performance from p-value thesholding:

Zheutlin AB, Dennis J, Karlsson Linnér R, Moscati A, Restrepo N, Straub P, et al. Penetrance and Pleiotropy of Polygenic Risk Scores for Schizophrenia in 106,160 Patients Across Four Health Care Systems. Am J Psychiatry 2019;176:846–55.

Can the authors justify their motivation for including PRS generated from all p-value threshold in the analysis and clarify this in the methods?

Author response: We used spectral decomposition of non-parametric correlations between PRS and the number of PNC phenotypes tested (see Nyholt, et al. PMID: 14997420). This method resulted in correcting for 13,787 independent tests (described in lines 129-130).

Indeed, the selection criteria of p-value thresholds depends on the design of the study. We chose not to select a PRS threshold *a priori* because of the likelihood that ASD polygenicity overlaps with neurodevelopment to varying degrees (e.g., different ASD risk loci are optimal for predicting different neurodevelopmental domains). Zheutlin, et al. chose to include all variants in their PRS (p-threshold = 1) but more importantly they used a different approach to polygenic risk scoring than we employed here (called PRS-CS). The methodological differences between PRSice and PRS-CS stem from how continuous shrinkage is applied. Through simulation studies in Ge, et al. (PMID: 30992449) it was demonstrated that PRS-CS underperforms when the training sample is relatively small (discussed in Ge, et al. in relation to a binary GWAS with 34,600 samples). They go on to conclude that the GWAS samples sizes for most GWAS may be too small to accurately measure the shrinkage parameter (a critical prior applied to the polygenic scores).

3. The authors note that the ASD PRS association with PEITANG did not replicate in young probands. Can the heritability of PEITANG be tested the young proband group and compared to the older samples using snp-based approaches like GCTA?

Author response: SNP-based heritability of the Penn Emotion Identification Test in the PNC has been estimated previously. Robinson, et al. (PMID: 25023143) report a SNP heritability of 35.7% ± 9.3 for the latent factor of emotion identification, of which anger recognition is an index phenotype. Using the GREML Power Calculator, the PNC proband groups are underpowered to detect significant heritability of PEITANG consistent with the Robinson, et al. estimate.

4. Was male-sex associated with PEITAND and were any sex-PRS interactions observed?

Author response: Sex was associated with PEITANG (p=0.019) independently of ancestry principal components and age. This difference reflects slightly lower PEITANG scores in males (mean PEITANG=5.22±1.59) relative to females (mean PIETANG=5.33±1.58). We did covary for sex in our ASD PRS phenome-wide association tests. We discuss in the text that though we adjust for sex effects, there is evidence that more nuanced sex-differences likely play a role in the lack of replication in the YP group (lines 257-264).

5. How many ASD samples are present in the NPC cohort? If samples are present in the NPC, what is the PEITAND distribution in ASD cases compare to the remainder of the cohort.

Author response: There are 184 participants from the quality controlled PNC cohort who answered “yes” to the ASD question in the assessment (11 AP, 109 MP, and 64 YP). We have included power calculations in the main text describing insufficient power to include the ASD probands from the PNC in our assessment of ASD PRS versus PEITANG. We have explored the association between ASD PRS and PEITANG in 184 ASD individuals and indeed to not detect an association, though this was expected, as we were grossly underpowered to do so. This information has been added to the main text (lines 106-108 and lines 385-398).

6. In the discussion, the authors describe the association between the ASD and PEITANG in the context of the ASD phenotype. However, a stronger association with PEITANG was observed with the schizophrenia PRS, indicating that increased recognition of angry faces may be a common feature of the genetic risk for neuropsychiatric conditions and not ASD-specific. Can the authors comment on this alternative explanation in their manuscript? The discussion of systematizing reads as conjecture without evidence and should be reduced in the text.

Author response: We investigated the independence of the ASD PRS association with PEITANG trait with respect to other psychiatric traits. Our findings support that the effect of ASD and SCZ PRS on this trait is independent. The strongest association observed in SCZ PRS analysis is likely due to the fact that the SCZ GWAS is much more powerful than the ASD GWAS. We clarified this point in the discussion section (lines 233-240).

We have reduced our discussion of systemizing theory.

7. Are there reports of PheWAS of the ASD PRS in the UK Biobank? If so, this would be an obvious study of an adult population for comparison with the results in adolescents here.

Author response: To our knowledge there are no PheWAS of ASD PRS in the UK Biobank; however, this would answer a much different question than the one sought out here. The UKB represents a wide array of diseases, environmental factors, and lifestyle factors. Here we focus on detailed neurodevelopmental features that are not available in the UK Biobank for comparison. We added this limitation in the discussion section (lines 285-288).
